# Medication-related perceptions of children and adolescents with severe asthma and moderate-to-severe atopic dermatitis: a non-interventional exploratory study

**DOI:** 10.1186/s13223-025-00961-8

**Published:** 2025-04-07

**Authors:** Markus Herzig, Maike vom Hove, Astrid Bertsche, Tobias Lipek, Wieland Kiess, Thilo Bertsche, Freerk Prenzel, Martina Patrizia Neininger

**Affiliations:** 1 Clinical Pharmacy, Institute of Pharmacy, Medical Faculty, Leipzig University and Drug Safety Center, Leipzig University and University Hospital, Bruederstrasse 32, D-04103 Leipzig, Germany; 2 Leipzig Interdisciplinary Center for Allergy (LICA), Liebigstraße 20a, 04103 Leipzig, Germany; 3 Center for Pediatric Research, University Hospital for Children and Adolescents, Liebigstrasse 20a, 04103 Leipzig, Germany; 4 Division of Neuropediatrics, University Hospital for Children and Adolescents, Ferdinand-Sauerbruch-Strasse 1, 17475 Greifswald, Germany; 5 German Center for Child and Adolescent Health (DZKJ), Partner Site Greifswald/Rostock, Ellernholzstraße 1-2, 17487 Greifswald, Germany

**Keywords:** Pediatrics, Asthma, Dermatitis, atopic, Antibodies, monoclonal, Drug therapy

## Abstract

**Background:**

Severe asthma and moderate-to-severe atopic dermatitis can significantly impact the lives of children and adolescents. However, real-world data on pediatric patients’ perceptions of their medication are limited.

**Methods:**

This non-interventional cross-sectional study at a university hospital explored patients’ perceptions. We included patients aged between 6 and 17 with severe asthma and/or moderate-to-severe atopic dermatitis. For patients treated with dupilumab, a minimum dupilumab treatment duration of 16 weeks was required. We conducted one structured interview per patient, based on a questionnaire consisting of open questions and ratings on 6-point Likert scales (response scale range: “0: not at all” to “5: very strongly”).

**Results:**

The study included 57 participants (severe asthma: *n* = 31; moderate-to-severe atopic dermatitis: *n* = 21; both: *n* = 5) who reported a “rather moderate” burden of asthma (median: 2; Q25/Q75: 0.3/2.8) or atopic dermatitis (3; 1.5/3.5). They experienced their current medications as “rather helpful” (asthma: 4; 3/5; atopic dermatitis: 4; 3/5). Twelve of the participants (21%) reported refusing to take their medication because of reluctance, but all resumed treatment. All participants receiving dupilumab therapy (*n* = 16) reported an improvement in their disease within a maximum of 2.5 months after starting treatment. The median fear of injection decreased from 3 (0/5) before the first injection to 0.5 (0/1) at the time of the survey.

**Conclusions:**

In this real-world, interview-based study, we found that pediatric patients perceived treatment as highly beneficial for asthma and atopic dermatitis. Furthermore, pediatric patients seemed to respond well to dupilumab therapy in terms of both disease improvement and less fear of injection.

**Trial registration:**

DRKSID DRKS00028092

**Supplementary Information:**

The online version contains supplementary material available at 10.1186/s13223-025-00961-8.

## Background

Asthma and atopic dermatitis are the most common chronic diseases in childhood and adolescence [1, 2]. Both conditions are classified as atopic diseases, characterized by a genetic predisposition to develop allergic reactions due to an overactive immune system response to allergens. They share overlapping pathogenetic mechanisms [3]. In their severe forms, these diseases can seriously affect the quality of life of children and adolescents and cause a high burden of disease [4–9]. Various medications are used to treat both diseases, with corticosteroids as the primary drug group [10–13]. However, adherence to topical corticosteroids for atopic dermatitis is compromised by several factors, including lack of medication efficacy, inconvenience and fear of adverse drug reactions [14]. A similar pattern of non-adherence to inhaled corticosteroids is commonly seen in asthma; fear of adverse drug reactions was identified as a reason for low adherence in 49–71% of patients, leading to decreased asthma control [15, 16]. Research suggests that adolescents prefer drugs with a faster and more persistent effect [17]. Monoclonal antibodies can offer this; most antibodies do not require daily administration [12, 18]. Dupilumab is an anti-IL-4Rα monoclonal antibody that inhibits the effects of interleukin 4 and interleukin 13. It is used for atopic dermatitis, asthma, chronic rhinosinusitis with nasal polyposis and eosinophilic esophagitis [19]. In clinical trials, standardized quality-of-life questionnaires and clinical disease assessment tools showed improvements for both diseases in ages 6 to < 18 years [20–23]. Dupilumab has been associated with an improvement in symptoms and quality of life [24–26]. However, only a limited number of pediatric patients have been assessed in real-world observational studies outside the controlled environment of phase 3 trials [27–32]. Those studies were limited to standardized questionnaires that assessed quality of life but did not specifically elaborate on how the disease affects children’s everyday lives and whether they were satisfied with their medication.

The extent to which fear of the injection represents an obstacle to treatment for children and adolescents has not yet been investigated. However, it is related to adherence and treatment success. Although other studies suggest that anxiety decreases with the duration of injection treatment [33], specific studies with dupilumab are still lacking.

Given the paucity of data, we conducted a non-interventional cross-sectional exploratory study to examine children’s and adolescents’ perceptions of the impact of their severe asthma and/or moderate-to-severe atopic dermatitis on various aspects of daily life under current treatment. We explored their perceptions of their medications and assessed their quality of life. Additionally, we aimed to investigate the participants’ subjective views on dupilumab treatment, specifically the onset of action and perceived fear of injection. Furthermore, we assessed the participants’ experience of changes in their allergies because previous reports have indicated a potential positive effect of dupilumab [34].

## Methods

### Study design

This survey of children and adolescents was a non-interventional cross-sectional explorative monocentric study (DRKS-ID: DRKS00028092; Universal Trial Number: U1111-1274-0966). The Ethics Committee of the Medical Faculty of Leipzig University granted approval (Reg. no: 050/22-ek). The participants were enrolled in the pediatric division of Leipzig Interdisciplinary Center for Allergy, located at the University Hospital for Children and Adolescents, between 02 May 2022 and 30 September 2023. Patients and their parents were approached during their appointment at the outpatient clinic. Comprehensive patient information was provided to the patients and their parents. Written informed consent was obtained from parents and from adolescents aged 14 years and older. Participation was voluntary.

### Inclusion criteria

Patients with severe asthma and/or moderate-to-severe atopic dermatitis were included. Patients with severe asthma were required to receive an add-on treatment to their basic therapy, consisting of at least 6 months of long-acting anticholinergic (LAMA) or a monoclonal antibody and/or the need for a high daily dose of inhaled corticosteroids, based on the “German national guideline for asthma” [10]. Patients with moderate-to-severe atopic dermatitis had to have a diagnosis based on the SCORing Atopic Dermatitis (SCORAD) with a score of at least 25, according to the “Consensus-based European guidelines for treatment of atopic eczema (atopic dermatitis) in adults and children” [11, 35]. Additional inclusion criteria were an age of at least 6 and less than 18 years, sufficient communicative abilities and the intellectual ability to understand and answer the questions. Participants receiving dupilumab were also required to have received it for at least 16 weeks, based on the patient record.

### Exclusion criteria

Current treatment with the anti-IgE-antibody omalizumab was an exclusion criterion because only two patients in our study received it and would be eligible for the analysis. This was identified as a potential risk of bias.

### Data collection based on a structured interview

An expert panel of clinical pharmacists and pediatricians developed a structured questionnaire to ensure consistent data quality across participants. A trained pharmacist conducted the interviews with the children and adolescents in a protected area of the department. The questionnaire consisted of two parts (Additional File 1). Part A was used to collect data from all participants about their perceptions of their asthma and/or atopic dermatitis and the related treatment. Part B of the questionnaire included only specific questions about treatment with dupilumab and was administered only to participants treated with dupilumab. Participants’ perceptions before dupilumab therapy were obtained by recall at the time of the interview and not before the first administration. The questionnaires included open questions that permitted multiple responses, as well as yes/no inquiries and questions that required participants to rate their answers on one of the pre-defined 6-point Likert scales, which were always visible (response scale):

0 - not at all; 1 - very little; 2 - rather little; 3 - moderately; 4 - rather strongly; 5 - very strongly.

0 - not at all; 1 - very little; 2 - rather little; 3 - moderately; 4 - rather a lot; 5 - very much.

After the interview, all children and adolescents with atopic dermatitis self-administered the Children’s Dermatology Life Quality Index (CDLQI) [36]. Those with asthma self-administered the Paediatric Asthma Quality of Life Questionnaire Standardised (PAQLQ(S)) [37]. The PAQLQ(S) is an instrument used to measure the impact of asthma on a child’s quality of life by assessing their symptoms, activity limitations, and emotional response to their condition across 23 questions in three domains [37]. The PAQLQ(S) total score, obtained by averaging the scores of the individual questions, ranges from 1 to 7, with a higher score indicating less impact on quality of life. The CDQLI total score ranges from 0 to 30, with a lower score indicating less impact on quality of life.

If the participants met the inclusion criteria for both asthma and atopic dermatitis, data were collected for both diseases. Supplemental patient data were extracted from the patient records on medication use, SCORAD, Asthma Control Test (ACT), the treatment duration of antibody therapy and diagnoses. Data on severe pulmonary exacerbations (PEx) were extracted from the patient records and categorized as follows: emergency therapy with short-acting beta2-agonist plus systemic corticosteroids or multiple uses of short-acting beta2-agonists on three consecutive days, presentation to the emergency department, or hospitalization due to asthma. If another antibody was administered before dupilumab therapy, e.g., omalizumab, the data from the SCORAD, ACT and PEx before the initial antibody administration were utilized in the analysis.

### Statistical analysis

We used IBM SPSS Statistics 29.0.1.0 (IBM, Armonk, NY, USA) for statistical analysis. First, we considered the entire study population. We determined the median, Q25/Q75 and min/max for the children’s and adolescents’ responses on the response scales and absolute and relative frequencies for nominal data. PAQLQ(S) and CDLQI results are reported as median, Q25/Q75 and min/max. In the second step, we analyzed the data from participants receiving dupilumab therapy to compare individual patients’ parameters before and after dupilumab treatment initiation. We did not compare dupilumab users to non-users, so matching was not required. We tested for normal distribution to ascertain the appropriateness of the applied statistical tests. As the values of SCORAD were normally distributed, we used a paired t-test to compare before and after dupilumab treatment initiation. As non-normal distribution was given for the ACT, pEX and the responses on the Likert scales, two-tailed Wilcoxon tests for paired data were used. Effect sizes were reported according to Cohen’s classification (small effect d ≥ 0.2/|r|≥0.1; medium effect d ≥ 0.5/|r|≥0.3; large effect d ≥ 0.8/|r|≥0.5; d: standardized mean difference;|r|: Pearson correlation coefficient) [38]. Post hoc power calculations were performed using G*Power 3.1.9.7 [39]. A *p*-value of ≤ 0.05 was considered to indicate significance.

## Results

### Characteristics of the study population

**Fig. 1:**
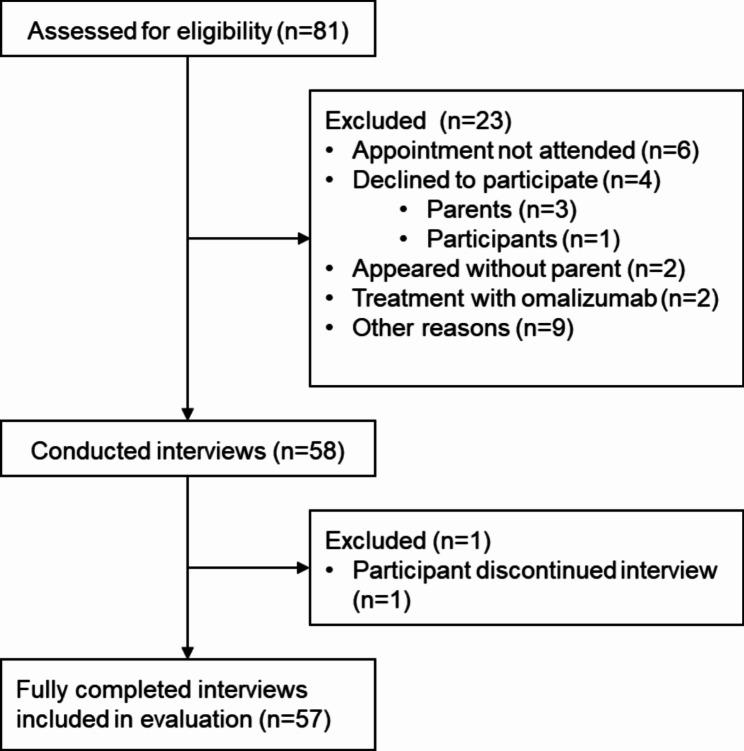
Patient enrolment flowchart

Our study enrolled 57 children and adolescents (Fig. 1). Patient characteristics are presented in Table 1. Sixteen participants received treatment with dupilumab. The median time between the start of dupilumab therapy and the interview was 10 months (Q25/Q75: 4/19; min/max: 4/50).

**Table 1:** Participants’ characteristics with and without dupilumab treatment at the time of the interview

	No treatment with dupilumab	Treatment with dupilumab	Total
**Number of participants**	**41**	**16**	**57**
**Median age** (Q25/Q75; min/max) [years]	10.0 (9.0/11.5; 7.0/16.0)	12.0 (9.0/14.0; 6.0/16.0)	11.0 (9.0/13.0; 6.0/16.0)
**Female** [n (%)]	18 (44%)	9 (56%)	27 (47%)
**Current educational status** [n (%)]			
Preschool	0 (0%)	1 (6%)	1 (2%)
Elementary school	23 (56%)	5 (31%)	28 (49%)
Secondary School	9 (22%)	2 (13%)	11 (19%)
Grammar School	9 (22%)	7 (44%)	16 (28%)
Special needs school	0 (0%)	1 (6%)	1 (2%)
**Disease** [n (%)]			
Severe Asthma	30 (73%)	1 (6%)	31 (54%)
Moderate-to-severe atopic dermatitis	10 (24%)	11 (69%)	21 (37%)
Both diseases	1 (2%)	4 (25%)	5 (9%)

### Quality of life and pediatric perspective of medication use

The participant-reported PAQLQ(S) score for the entire study population was 6.3 (median; Q25/Q75: 5.7/6.7; min/max: 3.7/7.0; Table S1 [Additional File 2]), and the median CDQLI score was 3 (Q25/Q75: 1/5; min/max: 0/17), suggesting a low impact of the participants’ asthma or atopic dermatitis on their quality of life at the time of the study. Table 2 shows the perceived impact of the diseases on various aspects of pediatric patients’ lives.

**Table 2:** Perceived impact of the diseases on various aspects of the pediatric patients’ lives. Participants’ responses on the likert scales are given as median, Q25/Q75, min/max. A total of 36 participants with severe asthma and 26 participants with moderate-to-severe atopic dermatitis were surveyed. Response scale: 0 - not at all; 1 - very little; 2 - rather little; 3 - moderately; 4 - rather strongly; 5 - very strongly

Question	Asthma	Atopic dermatitis
How much does your asthma/ atopic dermatitis bother you overall?	N: 36 Median: 2 Q25/Q75: 0.3/2.8 Min/Max: 0/4	N: 25* Median: 3 Q25/Q75: 1.5/3.5 Min/Max: 0/5
How much does your asthma/ atopic dermatitis bother you in your leisure time, e.g. when you are playing, pursuing your hobbies, or doing something outdoors?	N: 36 Median: 1 Q25/Q75: 0/2.8 Min/Max: 0/5	N: 25* Median: 1 Q25/Q75: 0/3 Min/Max: 0/5
How much does your asthma/ atopic dermatitis bother you when you are at school, e.g. in the classroom (not including physical education)?	N: 36 Median: 0 Q25/Q75: 0/0 Min/Max: 0/3	N: 24* Median: 1 Q25/Q75: 0/2 Min/Max: 0/4
How much does your asthma/ atopic dermatitis disturb you when you sleep?	N: 35* Median: 0 Q25/Q75: 0/0 Min/Max: 0/3	N: 26 Median: 1 Q25/Q75: 0/3 Min/Max: 0/5
Do you have the feeling that you are excluded by others because of your asthma/ atopic dermatitis?	N: 36 Median: 0 Q25/Q75: 0/0 Min/Max: 0/3	N: 25* Median: 0 Q25/Q75: 0/0 Min/Max: 0/1

*The remaining participant(s) did not provide a response

Participants with severe asthma and participants with moderate-to-severe atopic dermatitis responded “rather a lot” to the question “Overall, how much do your current medications help you with your asthma/ atopic dermatitis?” (median: 4; Q25/Q75: 3/5; min/max: 1/5 and median: 4; Q25/Q75: 3/5; min/max: 2/5, respectively).

Across the entire cohort of included patients, children and adolescents reported that using medications in everyday life bothered them “rather little” (median 2; Q25/Q75: 0/3; min/max: 0/5).

As shown in Table 3, 39/57 (68%) participants indicated that they had not used their medication as prescribed for various reasons. All participants resumed treatment (Table 3).

**Table 3:** Reasons and frequency for non-use of medications and reasons for subsequent resumption of use as reported by 39/57 (68%) participants. Multiple answers were possible

Reason for non-use	Frequency of the reason for non-use [*n* (%)]	Reason why participants used their medication again
Missed a dose	29/57 (51%)	“Thought about it again” “I immediately noticed a deterioration” 27 participants without specification
Refusing to take medication	12/57 (21%)	“Because I got worse again; because of corona” “Because I have to” “So that it gets better afterwards; so that no more medication is needed later” “Hands were torn open and bloody; because it doesn’t get better without it” “Because I thought it would help, I started again myself” “Because dad said that otherwise I would have to go back to hospital” “Because the doctor said so” “I didn’t want to take it [the medication] because it was still unknown” [referring to the short period after marketing authorization] “Because it got worse” 3 participants without specification
Adverse drug reaction	2/57 (4%)	“Because the allergy got worse” 1 participant without specification
Disgusted by the medication	1/57 (2%)	“Taste doesn’t matter after all”
No effect noticeable	1/57 (2%)	“Because it doesn’t get any better without it”
“I thought the spray was already gone”	1/57 (2%)	“The spray was still full”
“I tried out whether it works without it”	1/57 (2%)	“Because then it got worse again”
Not specified	1/57 (2%)	Not specified

Of the 57 participants, 13 (23%) reported having experienced at least one adverse drug reaction (Table 4).

**Table 4:** Experienced adverse drug reactions reported by participants and the intensity with which participants felt bothered by the respective adverse drug reaction. Multiple answers were possible. Response scale: 0 - not at all; 1 - very little; 2 - rather little; 3 - moderately; 4 - rather strongly; 5 - very strongly

Active ingredient	Adverse drug reaction (ADR)	Intensity with which the participants felt bothered by the ADR
“All creams”	Stinging skin	rather strongly (1 participant)
“Asthma spray”	Increased thirst	not at all (1 participant)
Cetirizine	Tiredness	not at all (1 participant)
“Cream”	Stinging skin	very strongly (2 participants)
Dupilumab	Herpes labialis	rather little (1 participant)
Dupilumab	Eye redness	not at all (1 participant)
Dupilumab	Conjunctivitis	very strongly (1 participant)
Dupilumab	Redness at the injection site	rather little (1 participant)
Dupilumab	Pain at the injection site	rather strongly (1 participant) very strongly (1 participant)
Fluticasone	Blurred vision	very little (1 participant)
Fluticasone/Formoterol	Vertigo	moderately (1 participant)
Loteprednol	Burning eyes	very strongly (1 participant)
Salbutamol	Trembling	rather little (1 participant)
Sublingual immunotherapy (house dust mite)	Itching when eating an apple Itching	not at all (1 participant) rather little (1 participant)

### ACT, PEx and SCORAD in dupilumab participants

The ACT showed an improvement in asthma control between the initiation of dupilumab treatment and the interview time (*N* = 5/5; median_Initiation_: 15, Q25/Q75_Initiation_: 12.5/17; median_Interview_: 22, Q25/Q75_Interview_: 20/25; *p* = 0.043;|r|=0.90; power > 0.99). In addition, the number of PEx episodes per year tended to decrease (*N* = 5/5; median_Initiation_: 2, Q25/Q75_Initiation_: 0.5/9; median_Interview_: 0, Q25/Q75_Interview_: 0/0.5; *p* = 0.068, n.s.;|r|=0.82; power = 0.29).

**Fig. 2:**
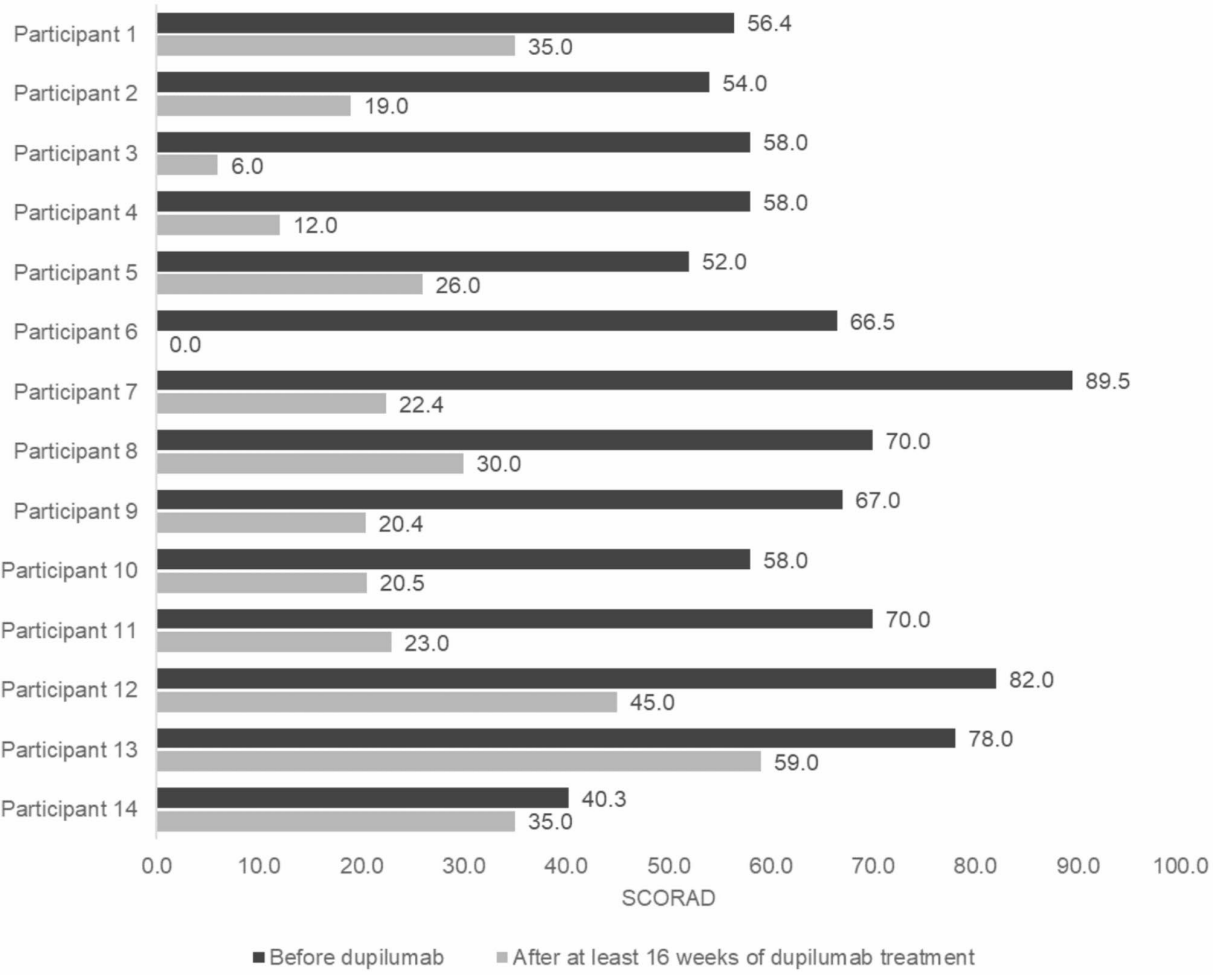
Participants’ individual SCORing Atopic Dermatitis (SCORAD) values before initiating dupilumab treatment and at the time of the interview are shown. A SCORAD > 50 is defined as severe, 25–50 as moderate and < 25 as mild atopic dermatitis [40]. SCORAD data were available from 14 of 15 participants with atopic dermatitis and current dupilumab therapy

The SCORAD assessment of atopic dermatitis revealed an improvement between initiation and interview, with a mean reduction of 60.8% (*N* = 14/15; mean_Initiation_: 64.3, SD_Initiation_: 13.1; mean_Interview_: 25.2, SD_Interview_: 15.3; *p* < 0.001; d: 2.247; power > 0.99; Fig. 2).

### Children’s and adolescents’ perceptions of their dupilumab therapy

**Fig. 3:**
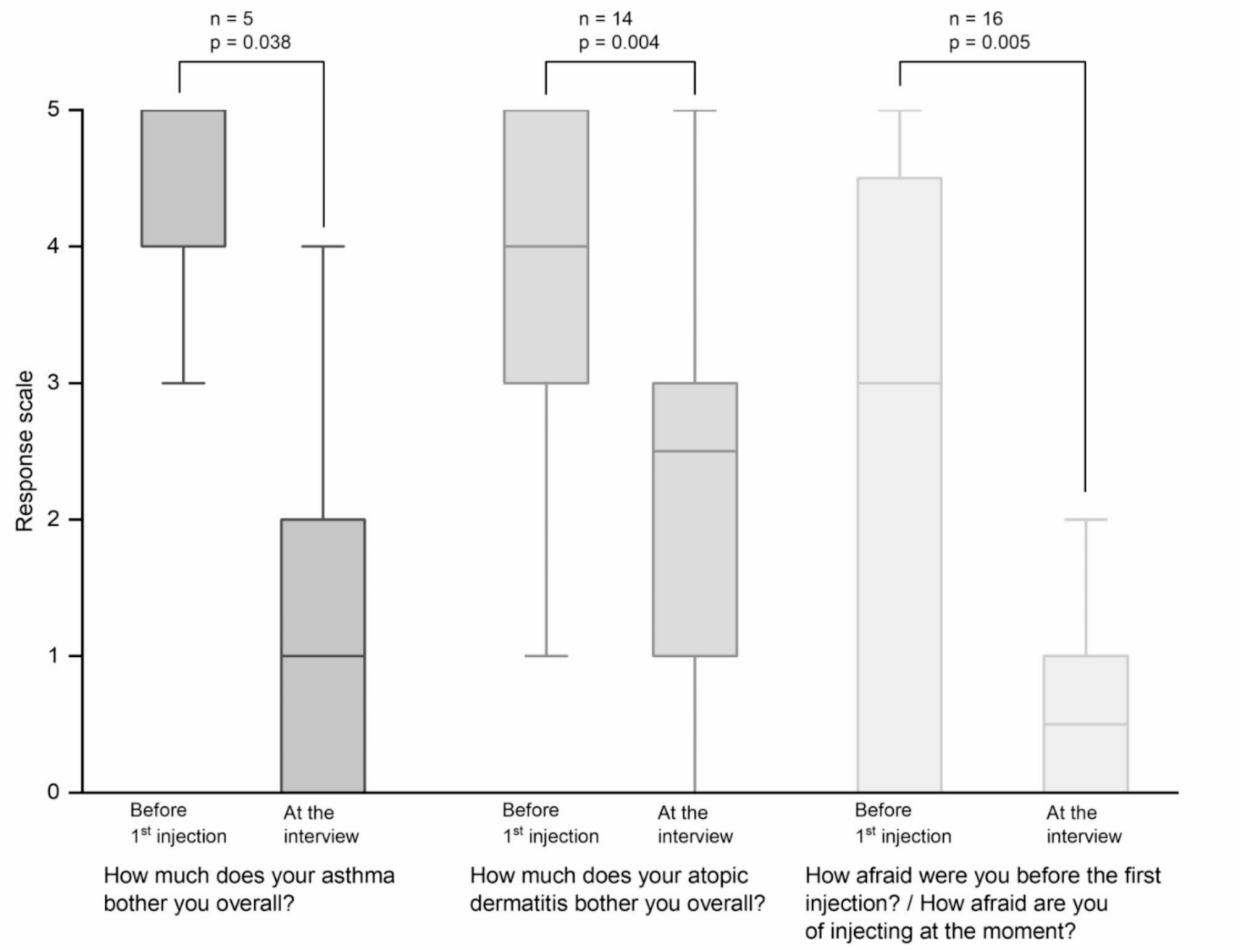
Participants’ perceptions of their diseases before and during dupilumab therapy (i.e., at the time of the interview). Data from 16 participants receiving dupilumab therapy for at least 16 weeks were analyzed. Boxplots show median, interquartile ranges (Q25/Q75) and whiskers extending to 1.5x interquartile range (Response Scale: 0 - not at all; 1 - very little; 2 - rather little; 3 - moderately; 4 - rather strongly; 5 - very strongly)

All participants receiving dupilumab reported an improvement in both asthma (5/5) and atopic dermatitis (15/15) since their first dose of dupilumab. Two participants reported an improvement in their asthma within 1 month, 1 participant within 1.5 months, 1 participant within 2 months and 1 participant did not specify when. For atopic dermatitis, the median time for participants to achieve improvement was 1.0 month (*n* = 14/15; Q25/Q75: 1.0/1.6; min/max: 0.3/2.5). As shown in Fig. 3, participants reported they were less bothered by their asthma (*p* = 0.038;|r|=0.93; power > 0.99) or atopic dermatitis (*p* = 0.004;|r|=0.76; power > 0.99) at the time of the interview compared to before initiating dupilumab treatment.

Participants reported that they were more afraid of the injection before the first injection than at the time of the interview (*p* = 0.005;|r|=0.70; power > 0.99), i.e., after several injections (Fig. 3).

### Children’s and adolescents’ perceptions of changes in allergies attributed to dupilumab

Of the 16 children and adolescents surveyed using dupilumab, 14 (88%) reported having at least one allergy. Of these 14, 7 (50%) observed improvement in their reactions to various allergens after initiating dupilumab treatment, including hazelnuts, nuts, house dust mites, animal dander, potatoes, cats, birch, alder, herbs, pears, cinnamon, apples, wheat, and cow’s milk.

## Discussion

This study suggests that the participants with treated asthma and atopic dermatitis had a high quality of life and a positive perception of their treatment, regardless of the type of therapy used. Additionally, those receiving dupilumab treatment reported an improvement in their disease after one to two months. This finding is supported by clinical disease assessment tools, such as the SCORAD, and self-reported positive perceptions. Furthermore, patients also reported a reduction in their fear of injections. Half of the patients with allergies reported an improvement in allergy symptoms.

### Quality of life and the perception of disease and therapy

The impact of asthma and atopic dermatitis on the quality of life of children and adolescents underscores the utmost importance of adequate treatment [4–9, 41]. Our analysis of patient-reported data from the PAQLQ(S) and the CDLQI indicated a high overall quality of life in the enrolled participants. Similarly, the participants seemed generally satisfied with the effectiveness of their medication. Our data suggest that they perceived their disease as having a relatively low impact on their daily lives under current treatment. Notably, more than half of all participants reported an incident of non-use of medication, e.g., missing a dose, refusing to take medication, or discontinuing due to perceived improvement in disease. However, they all resumed their therapy, primarily due to a noticeable worsening of symptoms, suggesting that the high quality of life of the included participants was owing to effective treatment. Particularly, participants with severe asthma and/or moderate-to-severe atopic dermatitis seemed to benefit from dupilumab treatment, as they reported a high quality of life. This is remarkable, as these diseases typically impact quality of life [4–9]. For instance, the most common issues in atopic dermatitis are itching, sleep disturbances, teasing, bullying, and exclusion from social interactions [42, 43]. As reported by the children and adolescents in our study, the comprehensive improvement in symptoms and exacerbations underline the potential to possibly reduce such problems and thus positively affect patients’ quality of life. This finding aligns with numerous studies demonstrating that dupilumab positively impacts both symptoms and quality of life in children and adolescents with severe asthma and moderate-to-severe atopic dermatitis [24–26, 32]. Furthermore, it is noteworthy that the surveyed patients seemed generally satisfied with their therapy even though treating atopic dermatitis often requires topical corticosteroids, emollients and moisturizers, which are frequently not adhered to due to poor user-friendliness [17].

As mentioned, bullying is a common issue in atopic dermatitis, but it is also a significant concern in asthma [44, 45]. A study showed that children and adolescents with asthma were more likely to experience bullying than the general population [45]. In our study, almost none of the treated patients with asthma and/or atopic dermatitis reported being affected by bullying. Thus, given the potential impact on social participation, it is crucial to provide effective therapies for asthma and atopic dermatitis, irrespective of whether these are conventional treatments or monoclonal antibodies.

### Perception of the participants on dupilumab therapy

We observed a mean reduction in SCORAD of 60.8% with a large effect size, and all participants achieved a SCORAD of less than 50 after the initiation of dupilumab treatment. Similar outcomes were reported in the phase 3 LIBERTY AD PEDS randomized controlled trial in children aged 6 to < 12 years and the LIBERTY AD ADOL study, which included adolescents aged 12 to < 18 years [20, 21, 23]. Our real-world data support that improvements in moderate-to-severe atopic dermatitis can also be achieved outside the controlled environment of phase 3 trials. This is consistent with other studies that showed similar SCORAD reduction results [27, 28].

In our study, standardized quality of life assessments using the PAQLQ(S) and CDLQI suggested a high disease-related quality of life for dupilumab patients, which is consistent with results from the dupilumab approval studies using the same questionnaires [20, 21, 46, 47]. The recent VOYAGE study on quality of life in asthma also demonstrated that children aged 6 to 11 years with moderate-to-severe asthma had improved PAQLQ(S) scores in the dupilumab group compared to the placebo group [24].

In our study, the improvement in disease severity was not only reflected in the children’s and adolescents’ clinical scores but was also discernible in the self-reported experiences of the participants. The median perceived response time to dupilumab treatment was between 1 and 1.3 months. Furthermore, the children and adolescents reported that as the injections became a routine part of their treatment, they experienced minimal or no fear of injection. Similar results were reported in a study of injection anxiety in children and adolescents receiving subcutaneous immunotherapy for allergy, which showed that injection anxiety decreased with duration of use [33]. Consequently, fear of injections should be considered in the treatment decision but should not be an absolute reason for deciding against a potentially effective antibody treatment.

In summary, these findings suggest that there is significant potential for improvement in disease burden after several weeks of dupilumab treatment, with a concomitant improvement in quality of life and a reduction of fear of injection. In addition, half of the participants with allergies reported an improvement in their allergy symptoms. Consequently, these findings support the current guideline recommendations to initiate dupilumab for treating severe asthma and moderate-to-severe atopic dermatitis in children and adolescents [13, 48, 49]. Thus, dupilumab may also be a potential treatment option for allergic conditions in children and adolescents, but further research is required.

### Strengths and limitations

Our study provides an explorative insight into the perceptions of children and adolescents with severe asthma and moderate-to-severe atopic dermatitis. We obtained the subjective views of pediatric patients on their disease and therapy, particularly dupilumab. As a result, the data allow for a better understanding of this specific patient population.

Although restrictive inclusion criteria can benefit the study of specific patient populations, they can also risk limiting participant enrollment. Only 57 participants were recruited in this study, 16 of whom received dupilumab treatment. However, statistical analyses based on Cohen’s calculations indicated large effect sizes, suggesting that the sample size was sufficient. This finding was further supported by the high values observed in the post hoc power calculations. Nevertheless, the conclusions drawn from these results should be interpreted cautiously. Furthermore, it is possible that a recall bias may have influenced the reported perception of the disease and the fear of injections before dupilumab therapy. However, at any time during the interview, participants had the option to indicate if they were not sure or could not remember.

Furthermore, the limited number of potential participants made it impractical to implement a randomized study design, which would permit direct comparisons between patients with and without dupilumab therapy while reducing potential confounding factors. However, routine data allowed us to capture the actual care setting and treatment, including the diversity of treatments used in daily practice. This gave us valuable insights into real-world patient care instead of the controlled environment of clinical trials.

## Conclusions

This exploratory study examined children’s and adolescents’ perspectives on medication use for severe asthma and/or moderate-to-severe atopic dermatitis. Patients reported a high quality of life and positive perceptions of their treatment. Dupilumab therapy was associated with improvements in ACT, PEx, and SCORAD scores, as well as symptom relief within weeks. Initial fears of injections appeared to diminish after a few administrations. While further research is needed to confirm these findings and explore long-term effects, our data highlight the potential benefits of dupilumab in pediatric clinical care. Importantly, the initial fear of injections should not preclude its use, as most patients overcome this fear during treatment.

## Electronic supplementary material

Below is the link to the electronic supplementary material.

Supplementary Material 1: Additional File 1: Questionnaire for the interview

Supplementary Material 2: Additional File 2: Results of the questionnaires Children’s Dermatology Life Quality Index (CDLQI) and Paediatric Asthma Quality of Life Questionnaire Standardised (PAQLQ(S))

## Data Availability

The data that support the findings of this study are available on request from the corresponding author.

## Abbreviations

ACT: Asthma Control Test
CDLQI: Children’s Dermatology Life Quality Index
PAQLQ(S): Paediatric Asthma Quality of Life Questionnaire Standardised
PEx: Pulmonary exacerbations
SCORAD: SCORing Atopic Dermatitis
