# Supplementary material for: Medication-related perceptions of children and adolescents with severe asthma and moderate-to-severe atopic dermatitis: a non-interventional exploratory study
Source: Allergy Asthma Clin Immunol. 2025 Apr 7;21:16. doi: 10.1186/s13223-025-00961-8 (PMC11978019; doi:10.1186/s13223-025-00961-8)
Supplement: Supplementary file 1 — Supplementary Material 1: Additional File 1: Questionnaire for the interview [file 13223_2025_961_MOESM1_ESM.pdf]

Additional File 1

Medication-related perceptions of pediatrics with severe asthma and moderate-to-severe atopic dermatitis: A non-interventional exploratory study

Markus Herzig[a], Maike vom Hove[b,c], Astrid Bertsche[c,d,e], Tobias Lipek[b,c], Wieland Kiess[c], Thilo Bertsche[a*], Freerk Prenzel[b,c#], Martina Patrizia Neininger[a,d,e#]

#Shared senior authorship

[a] Clinical Pharmacy, Institute of Pharmacy, Medical Faculty, Leipzig University and Drug Safety Center, Leipzig University and University Hospital, Bruederstrasse 32, 04103 Leipzig, Germany

[b] Leipzig Interdisciplinary Center for Allergy (LICA), Liebigstrasse 20a, 04103 Leipzig, Germany

[c] University Hospital for Children and Adolescents, Center for Pediatric Research, Liebigstrasse 20a, 04103 Leipzig, Germany

[d] University Hospital for Children and Adolescents, Division of Neuropediatrics, Ferdinand-Sauerbruch-Strasse 1, 17475 Greifswald, Germany

[e] German Center for Child and Adolescent Health (DZKJ), partner site Greifswald/Rostock, Ellernholzstraße 1-2, 17487 Greifswald, Germany

***Corresponding author:**

Thilo Bertsche, Clinical Pharmacy, Institute of Pharmacy, Medical Faculty, Leipzig University and Drug Safety Center, Leipzig University and University Hospital, Bruederstrasse 32, D-04103 Leipzig, Germany; Tel.: +49-341-9711800, Fax: +49-341-9711813, e-mail: thilo.bertsche@uni-leipzig.de

Questionnaire for the interview. The interview was conducted in German, we translated the questionnaire into English for better comprehensibility.

Study questionnaire

Drug therapy in children and adolescents with severe asthma and/or moderate-to-severe atopic dermatitis

A survey of affected children and adolescents and their parents on satisfaction with drug therapy and drug use, with special consideration of immunotherapies

For some questions, participants were asked to rate their answers on pre-defined 6-point Likert scales. To facilitate the rating, the scales were printed out and presented to the participants:

0 - not at all; 1 - very little; 2 - rather little; 3 - moderately; 4 - rather strongly; 5 - very strongly

0 - not at all; 1 - very little; 2 - rather little; 3 - moderately; 4 - rather a lot; 5 - very much

Questions on asthma were only asked in patients with asthma, the same applies to atopic dermatitis. Patients with both conditions were asked for both conditions separately.

I would now like to ask you a few questions about your asthma or your atopic dermatitis, and about your medication. I will take a few notes on this. If you wish, you can read through them after we finished the interview.

If you don't understand a question, you are welcome to say so. I will then try to explain it to you in other words. If you don't want to answer a question, you can say so at any time.

Before we start with the questions about your asthma or your atopic dermatitis, I will ask you a few general questions to get to know you a little. What do you think, should we start or do you have any questions?

1. General questions

1.1 How old are you? ____________ years

1.2 Which school are you currently attending?

□ Preschool

□ Elementary school

□ Secondary school

□ Grammar school

□ Special needs school

□ Other: ___________________________

____

Interview part A

2. Questions about the disease in general

Now I would like to ask you a few questions about your asthma or your atopic dermatitis. You can answer some questions openly, but sometimes I will also give you possible answers or examples. Are you ready?

2.1 What is the name of your condition?

____

You have said that you have asthma and/or atopic dermatitis. I will now ask you a few questions about how the asthma/atopic dermatitis affects your everyday life. When answering, think about your asthma/atopic dermatitis in general, i.e. both phases when your asthma/atopic dermatitis is worse, as well as phases when you don't have any attacks/flare-ups. For those who have both: First, I will ask about asthma, then about atopic dermatitis.

[If asthma is present]:

2.2a How much does your asthma bother you overall?

□ not at all - □ very little - □ rather little - □ moderately - □ rather a lot - □ very much

2.3a How much does your asthma bother you in your leisure time, e.g. when you are playing, pursuing your hobbies, or doing something outdoors?

□ not at all - □ very little - □ rather little - □ moderately - □ rather a lot - □ very much

2.4a How much does your asthma bother you when you are at school, e.g. in the classroom (not including physical education)?

□ not at all - □ very little - □ rather little - □ moderately - □ rather a lot - □ very much

2.5a How much does your asthma disturb you when you sleep?

□ not at all - □ very little - □ rather little - □ moderately - □ rather a lot - □ very much

2.6a Do you have the feeling that you are excluded by others because of your asthma?

□ not at all - □ very little - □ rather little - □ moderately - □ rather strongly - □ very strongly

[If atopic dermatitis is present]:

2.2b How much does your atopic dermatitis bother you overall?

□ not at all - □ very little - □ rather little - □ moderately - □ rather a lot - □ very much

2.3b How much does your atopic dermatitis bother you in your leisure time, e.g. when you are playing, pursuing your hobbies, or doing something outdoors ?

□ not at all - □ very little - □ rather little - □ moderately - □ rather a lot - □ very much

2.4b How much does your atopic dermatitis bother you when you are at school, e.g. in the classroom (not including physical education)?

□ not at all - □ very little - □ rather little - □ moderately - □ rather a lot - □ very much

2.5b How much does your atopic dermatitis disturb you when you sleep?

□ not at all - □ very little - □ rather little - □ moderately - □ rather a lot - □ very much

2.6b Do you have the feeling that you are excluded by others because of your atopic dermatitis?

□ not at all - □ very little - □ rather little - □ moderately - □ rather strongly - □ very strongly

3. Questions on the use of medications and adverse drug reactions

3.1.1 Overall, how much do your current medications help you with your asthma?

□ not at all - □ very little - □ rather little - □ moderately - □ rather a lot - □ very much

3.1.2 Overall, how much do your current medications help you with your atopic dermatitis?

□ not at all - □ very little - □ rather little - □ moderately - □ rather a lot - □ very much

3.2 How much does it bother you that you have to use the medication at all in everyday life?

□ not at all - □ very little - □ rather little - □ moderately - □ rather a lot - □ very much

3.3 Have you ever stopped using your medication?

□ yes □ no □ don't know

[If yes] 3.3.1 What was the reason for this? (Open question, multiple answers possible).

3.3.2 Did you start taking the medication again afterwards?

□ yes □ no □ don't know

[If yes] 3.3.3 Why did you start taking the medication again? (Open question)

Do you know the word side effect?

[If no]: A side effect is an unintended reaction (e.g. stomach pain) of your body that occurs after taking a medication. Do you know what I mean?

3.4 Have you ever had a side effect from your current medication?

□ yes □ no □ don't know

[If yes] 3.4.1 From which medication? What side effect occurred?

1st medication: ____________________ Reported side effect:____________________

2nd medication: ____________________ Reported side effect:____________________

3rd medication: ____________________ Reported side effect:____________________

4th medication: ____________________ Reported side effect:____________________

[The following questions were asked for each side effect]:

3.4.2 How much did the side effect ___________________________ bother you?

□ not at all - □ very little - □ rather little - □ moderately - □ rather a lot - □ very much

Interview Part B

[Only to be performed if the patient is treated with a therapeutic antibody]

4. Questions on the use of and experience with therapeutic antibodies

Remember the time before you got dupilumab.

4.1.1 How much did the asthma bother you overall before you got dupilumab?

□ not at all - □ very little - □ rather little - □ moderately - □ rather a lot - □ very much

4.1.2 How much did the atopic dermatitis bother you overall before you got dupilumab?

□ not at all - □ very little - □ rather little - □ moderately - □ rather a lot - □ very much

4.2 How afraid were you before the first injection?

□ not at all - □ very little - □ rather little - □ moderately - □ rather a lot - □ very much

Now think back to the present moment.

4.3 How afraid are you of injecting at the moment?

□ not at all - □ very little - □ rather little - □ moderately - □ rather a lot - □ very much

4.4.1 Do you think your asthma has improved since you started using dupilumab?

□ yes □ no □ don't know

[If yes] 4.4.1 After how many months since starting the treatment has your asthma improved?

____

4.4.2 Do you think that your atopic dermatitis has improved since you started using dupilumab?

□ yes □ no □ don't know

[If yes] 4.4.1 After how many months since starting the treatment has your atopic dermatitis improved?

____

4.5 Do you have any allergies?

□ yes □ no □ don't know

[If yes] 4.5.1 What kind of allergies do you have?

____

4.5.2 Have you noticed that your allergies have improved since you started using dupilumab?

□ yes □ no □ don't know

[If yes; if several allergies] 4.5.3 For which type of allergy have you noticed an improvement?

____

You did a great job. Thank you very much for taking part in the survey.
