# Supplementary material for: Medication-related perceptions of children and adolescents with severe asthma and moderate-to-severe atopic dermatitis: a non-interventional exploratory study
Source: Allergy Asthma Clin Immunol. 2025 Apr 7;21:16. doi: 10.1186/s13223-025-00961-8 (PMC11978019; doi:10.1186/s13223-025-00961-8)
Supplement: Supplementary file 2 — Supplementary Material 2: Additional File 2: Results of the questionnaires Children’s Dermatology Life Quality Index (CDLQI) and Paediatric Asthma Quality of Life Questionnaire Standardised (PAQLQ(S)) [file 13223_2025_961_MOESM2_ESM.pdf]

Additional File 2

Medication-related perceptions of pediatrics with severe asthma and moderate-to-severe atopic dermatitis: A non-interventional exploratory study

Markus Herzig[a], Maike vom Hove[b,c], Astrid Bertsche[c,d,e], Tobias Lipek[b,c], Wieland Kiess[c], Thilo Bertsche[a*], Freerk Prenzel[b,c#], Martina Patrizia Neininger[a,d,e#]

#Shared senior authorship

[a] Clinical Pharmacy, Institute of Pharmacy, Medical Faculty, Leipzig University and Drug Safety Center, Leipzig University and University Hospital, Bruederstrasse 32, 04103 Leipzig, Germany

[b] Leipzig Interdisciplinary Center for Allergy (LICA), Liebigstrasse 20a, 04103 Leipzig, Germany

[c] University Hospital for Children and Adolescents, Center for Pediatric Research, Liebigstrasse 20a, 04103 Leipzig, Germany

[d] University Hospital for Children and Adolescents, Division of Neuropediatrics, Ferdinand-Sauerbruch-Strasse 1, 17475 Greifswald, Germany

[e] German Center for Child and Adolescent Health (DZKJ), partner site Greifswald/Rostock, Ellernholzstraße 1-2, 17487 Greifswald, Germany

***Corresponding author:**

Thilo Bertsche, Clinical Pharmacy, Institute of Pharmacy, Medical Faculty, Leipzig University and Drug Safety Center, Leipzig University and University Hospital, Bruederstrasse 32, D-04103 Leipzig, Germany; Tel.: +49-341-9711800, Fax: +49-341-9711813, e-mail: thilo.bertsche@uni-leipzig.de

Table S1: Results of the standardized questionnaires, the self-administered Paediatric Asthma Quality of Life Questionnaire Standardised (PAQLQ(S)), and the self-administered Children's Dermatology Life Quality Index (CDLQI)

	**Non-users of dupilumab**	**Users of dupilumab**	**Total**
PAQLQ(S)	Median: 6.3	Median: 5.5	Median: 6.3
	Q25/Q75: 5.8/6.7	Q25/Q75: 4.8/6.3	Q25/Q75: 5.7/6.7
	min/max: 3.7/7.0	min/max: 4.8/7.0	min/max: 3.7/7.0
CDLQI	Median: 5	Median: 2	Median: 3
	Q25/Q75: 2/6	Q25/Q75: 1/5	Q25/Q75: 1/5
	min/max: 1/9	min/max: 0/17	min/max: 0/17
